# Profiling of metabolome and bacterial community dynamics in ensiled *Medicago sativa* inoculated without or with *Lactobacillus plantarum* or *Lactobacillus buchneri*

**DOI:** 10.1038/s41598-017-18348-0

**Published:** 2018-01-10

**Authors:** X. S. Guo, W. C. Ke, W. R. Ding, L. M. Ding, D. M. Xu, W. W. Wang, P. Zhang, F. Y. Yang

**Affiliations:** 1 State Key Laboratory of Grassland and Agro-ecosystems, School of Life Sciences, Lanzhou University, Lanzhou, 730000 PR China; 2 Probiotics and Biological Feed Research Center, Lanzhou University, Lanzhou, 730000 PR China; 3 Institute of Grassland Science, College of Animal Science and Technology, China Agricultural University, Beijing, 100193 PR China

## Abstract

Using gas chromatography mass spectrometry and the PacBio single molecule with real-time sequencing technology (SMRT), we analyzed the detailed metabolomic profiles and microbial community dynamics involved in ensiled *Medicago sativa* (alfalfa) inoculated without or with the homofermenter *Lactobacillus plantarum* or heterofermenter *Lactobacillus buchneri*. Our results revealed that 280 substances and 102 different metabolites were present in ensiled alfalfa. Inoculation of *L. buchneri* led to remarkable up-accumulation in concentrations of 4-aminobutyric acid, some free amino acids, and polyols in ensiled alfalfa, whereas considerable down-accumulation in cadaverine and succinic acid were observed in *L. plantarum*-inoculated silages. Completely different microbial flora and their successions during ensiling were observed in the control and two types of inoculant-treated silages. Inoculation of the *L. plantarum* or *L. buchneri* alters the microbial composition dynamics of the ensiled forage in very different manners. Our study demonstrates that metabolomic profiling analysis provides a deep insight in metabolites in silage. Moreover, the PacBio SMRT method revealed the microbial composition and its succession during the ensiling process at the species level. This provides information regarding the microbial processes underlying silage formation and may contribute to target-based regulation methods to achieve high-quality silage production.

## Introduction

Ensiling is a traditional method of green fodder conservation involving lactic acid fermentation by epiphytic lactic acid bacteria (LAB) under anaerobic conditions. After airtight sealing, trapped oxygen is expended by plant and microbial respiration and the subsequent anaerobic environment terminates plant respiration, inhibits the growth of aerobic microorganisms, and creates favorable conditions for spontaneous fermentation by epiphytic LAB^1^. Because silage fermentation quality mainly depends on the microbial communities present and their succession as well as fermentative metabolites during ensiling^2^, a better understanding of the process underlying silage formation may provide a valuable scientific basis for improving the ensiling process and silage fermentation quality.

In the past decade, metagenomic approaches, such as denaturing gradient gel electrophoresis^3^ and terminal restriction fragment length polymorphism^1^, have been employed to track changes in microbial communities and identify dominant species in ensiled forages. However, these techniques only reflect a few of the most abundant operational taxonomic units (OTU) present^4^ and do not reveal detailed information regarding the composition of the complete microbial community^5^. Although metagenomic analysis by 454 high-throughput sequencing provided more comprehensive insight into the composition of the whole microbial community involved in grass ensiling^5^, this technique is restricted to genus-level identification because only a partial sequence of the 16S rRNA gene is evaluated. A recent metagenomic approach, the PacBio single molecule, in conjunction with real-time sequencing technology (SMRT) can be used to reveal the bacterial profile of target samples at the species level because it can generate long sequence reads^6^. Therefore, the SMRT sequencing platform is applicable for profiling ensiling communities.

Metabolites in silage, such as organic acids (lactic acid, acetic acid, propionic acid, butyric acid), ethanol, and 1,2-propanediol, are conventionally detected to evaluate the fermentation quality of ensiled forage. Production of lactic acid and other volatile fatty acids in ensiled forage not only ensures that the green fodder will be well-preserved by pH reduction, but also will prevent the growth of yeasts, molds, and other contaminating microorganisms during ensiling and improve aerobic stability after aerobic exposure. Previous studies showed that LAB can improve silage aerobic stability by producing different metabolites, such as acetic acid and 1,2-propanediol^7,8^, 3-phenyllactic acid and 3-hydroxydecanoic acid^9^, and a phenolic-related antibiotic^10^. These studies suggested that many other metabolites are present in ensiled forage in addition to the conventional metabolites commonly detected when assessing silage quality. In addition, LAB can produce a large number of metabolites during fermentation, such as amino acids, fatty acids, oligosaccharides, vitamins, small peptides, flavoring agents, and aromatic compounds^11^. Therefore, it can be speculated that many metabolites in ensiled forage have not been identified.

Currently, silage starter cultures are widely used to improve the ensiling process in terms of acid formation rate and aerobic stability. Based on the fermentation pattern, starter cultures are divided into homofermentative and heterofermentatvie cultures. Homofermentative cultures, such as *Lactobacillus plantarum*, *Pediococcus* species, and *Enterococcus faecium*, are often used to promote the production of lactic acid and decease pH and nutrients losses^7^. The heterofermentative LAB *Lactobacillus buchneri* was shown to inhibit yeast and mold growth by producing high concentrations of acetic acid^12–14^. Therefore, the two types of starter cultures use different approaches for directing fermentation in ensiled forage^15^. In this study, we comprehensively analyzed the metabolomic profiles and bacterial community dynamics using the PacBio SMRT method in ensiled alfalfa, using the homofermenter *L. plantarum* and heterofermenter *L. buchneri* for modulation.

## Results

### Fermentation profiles of ensiled forage

All silages were well preserved with no detectable propionic and butyric acids (Table 1). Inoculation of *L. plantarum* decreased the silage pH and increased the lactate concentration in ensiled alfalfa. As expected, *L. buchneri* increased the acetate concentration in alfalfa silage. Yeast and mold growth was inhibited, while the number of LAB increased after ensiling. Inoculation with *L. plantarum* and *L. buchneri* markedly decreased the NH_3_-N content in ensiled alfalfa.

**Table 1:** Fermentation, chemical and microbial compositions of ensiled alfalfa inoculated without or with *L. plantarum* or *L. buchneri* after 90 days of fermentation.

Item^1^	Alfalfa silage^2^			SEM	*P*-value
	Control	LP	LB		
DM, g/kg	403^a^	400^a^	414^b^	2.3	0.005
CP, g/kg DM	198	198	199	0.8	0.92
NDF, g/kg DM	448	427	401	12.0	0.35
ADF, g/kg DM	280	266	282	5.37	0.46
NH_3_-N, g/kg total N	153^c^	93^a^	111^b^	10.0	<0.0001
WSC, g/kg DM	5.4^c^	3.9^a^	4.4^b^	0.26	<0.0001
pH	5.2^b^	4.9^a^	5.2^b^	0.06	0.001
Lactate, g/kg DM	38.6^a^	68.4^c^	52.6^b^	6.01	0.016
Acetate, g/kg DM	11.6^a^	15.8^ab^	17.6^b^	1.26	0.092
Lactic acid bacteria, log_10_ cfu/g	7.7^b^	7.4^a^	7.9^c^	0.07	0.005
Yeasts, log_10_ cfu/g	2.8	2.8 <	2.7 <	0.06	0.907
Molds, log_10_ cfu/g	2.3	<2.0	<2.0	—	—

^a–c^Within a row, means without a common superscript letter differ (*P* < 0.05). SEM, standard error of means. ^1^DM, dry matter; CP, crude protein; NDF, neutral detergent fiber; ADF, acid detergent fiber; WSC, water soluble carbohydrate.

^2^LP, *Lactobacillus plantarum* treatment; LB, *Lactobacillus buchneri* treatment.

### Metabolomic profiles of alfalfa silage

**Figure 1:**
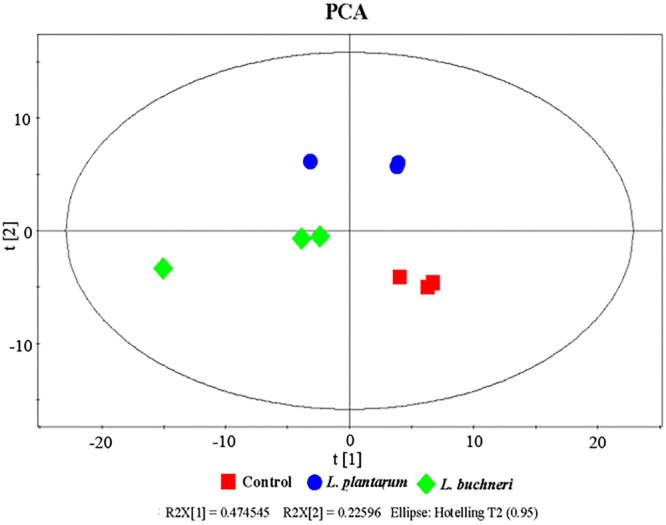
Principal component analysis (PCA) of metabolic profiles in alfalfa silage inoculated without (control) or with *L. plantarum* or *L. buchneri* (n = 3). Input data were the total mass of the signal integration area of each sample, and the signal integration area was normalized to 10,000 for each sample.

Based on the retention time and mass to charge ratio of total ions in the chromatograms of 9 silage samples (including control, *L. plantarum* and *L. buchneri*-inoculated groups with triplicate for each treatment and ensiled for 90 days), a total of 280 substances were detected, and 102 different metabolites were identified with their relative concentrations (File S1). According to principal component analysis (Figs 1 and S1), clear differences were observed between samples within treatments. Samples of control, *L. plantarum*, and *L. buchneri* were clearly separated by PC1, which represented 47.4% of variation among samples. PC2 distinguished the samples among the three treatments, explaining 22.6% of the variation. The contribution of metabolites to PC1 was dominated by amino acids (e.g., 4-aminobutyric acid, glutamic acid, leucine, glycine, threonine, and alanine) and polyols (e.g., mannitol, arabitol, erythritol, and threitol), whereas organic acids (e.g., 4-hydroxyphenylpropionic acid, ketomalonic acid, malonic acid, hexanoic acid, and lactic acid) were the major contributors to PC2 (Table S1).

Compared to the control silages, higher contents of 2,3-butandiol, adenine, threonine, tyrosine, aspartic acid, and lysine were observed in the *L. plantarum* and *L. buchneri* inoculated alfalfa silages (Table 2), whereas lower cadaverine, 2,3-butanediol, 2-aminobutyric acid, glutamic acid, and succinic acid were observed in the inoculants-treated silages. In addition, contents of cadaverine, 2-aminobutyric acid and succinic acid in *L. plantarum*-inoculated silages were lower than those in *L. buchneri*-inoculated silages. Inoculation of *L. buchneri* resulted in an up-accumulation in all amino acids, except for glutamic acid. Particularly, valine, aspartic acid, arabitol, erythritol, mannitol, aminomalonic acid, 4-aminobutyric acid, and glycerol were markedly higher in ensiled alfalfa than those in control silage. In *L. buchneri*-treated silages, 16 metabolites showed significantly higher contents than those in *L. plantarum* treated silages. The up-accumulated metabolites were mainly 2,3-butandiol, 2-aminobutyric acid, arabitol, erythritol, cadaverine, glycerol, succinic acid, threonine, tyrosine, valine, ornithine, and lysine.

**Table 2:** Relative concentration and fold-changes in major metabolites in alfalfa silages without or with inoculation of *L. plantarum* or *L. buchneri* after 90 days of ensiling.

Metabolite name	Relative concentration^1^			Fold-changes^2^		
	Control	*L. plantarum*	*L. buchneri*	log_2_^(P/C)^	log_2_^(B/C)^	log_2_^(P/B)^
2,3-Butandiol	86.59	170.9	340.38	0.981*	1.975*	0.993*
2-Aminobutyric acid	61.5	12.54	25.14	−2.294*	−1.291*	1.003*
4-Aminobutyric acid	112.41	169.14	272.19	0.589	1.276*	−0.686
Benzoic acid	43.86	56.89	55.81	0.375*	0.347	−0.027
Adenine	2.57	26.16	21.02	3.344*	3.029*	0.315
α–Hydroxyisobutyric acid	3.51	0.77	1.03	−2.188*	−1.762*	−0.423
Aminomalonic acid	5.39	8.81	10.33	0.709	0.938*	−0.228
Arabitol	4.36	6.21	21.43	0.511	2.298*	1.786*
Cadaverine	191.86	22.14	101.16	−3.11*	−0.924*	2.190*
Erythritol	10.44	10.49	39.07	0.006	1.902*	1.879*
Glycerol	190.26	184.08	301.31	−0.047	0.663*	0.711*
Inositol	31.74	42.04	40.93	0.406*	0.366	0.038
Ketomalonic acid	0.46	4.05	1.77	3.112*	1.925*	−1.188*
Malonic acid	2.15	10.89	7.68	2.337*	1.832*	−0.505*
Mannitol	0.43	0.47	3.94	0.103	3.184*	3.081*
Phenethylamine	3.62	0.27	1.16	−3.724*	0.762*	2.079*
Threitol	1.51	1.99	3.24	0.400*	1.101*	0.701*
Lactic acid	132	167	120	0.339*	−0.134	−0.473*
Succinic acid	158.28	82.96	111.72	−0.932*	−0.502*	0.429*
trans-Ferulic acid	3.91	1.69	5.34	−1.21	0.448	1.659*
Threonine	41.28	64.59	73.96	0.645*	0.841*	−0.195*
Tyrosine	3.23	16.76	98.42	4.774*	4.930*	−0.155*
Valine	162.1	172.45	219.74	0.089	0.439*	−0.349*
Ornithine	28.83	44.39	65.84	0.623	1.192*	−0.568
Lysine	12.15	38.26	50.24	1.654*	2.047*	−0.393*
β-Alanine	1.65	2.12	4.04	0.359	1.292*	−0.932
Aspartic acid	139.71	289.55	310.62	1.051*	1.153*	−0.101
Glutamic acid	76.86	39.42	28.59	−0.963*	−1.426*	0.463

^1^The relative concentration of each metabolite is an average of data from three biological replicates using GC-MS. ^2^The fold-changes were calculated using the formula log_2_^(X/Y)^. X and Y refer different treatments: C, control; B, *Lactobacillus buchneri* treatment; P, *Lactobacillus plantarum* treatment; * indicate significant (*P* < 0.05). The major metabolites were selected based on at least one of Fold- changes (log_2_^(P/C)^, log_2_^(B/C)^, log_2_^(P/B)^) contrast was statistically significant.

### Changes in bacterial microbiota dynamics during ensilage

Based on SMRT sequencing of the full-length 16S rRNA gene of silage bacteria, a total of 10,958 SMRT sequencing reads were obtained from the 13 samples. The chao1 curves showed that the sequence depth was adequate for all samples except for sample control-30 (Fig. S2). The α-diversity (Shannon index, Simpson index, Chao1 index), and number of observed species (Table 3) indicated low bacterial biodiversity in the present silage fermentation systems.

**Table 3:** Sequence and bacterial diversity of fresh forage and experimental treatment groups^1^. ^1^C, the control groups with different fermentation days; LB, *L. buchneri* inoculated groups with different fermentation days; LP, *L. plantarum* inoculated groups with different fermentation days. ^2^Good’s coverage: coverage is calculated as C = 1 − (*s*/*n*), where *s* is the number of unique OTUs and *n* is the number of individuals in the sample. This index gives a relative measure of how well the sample represents the larger environment.

	Number of reads	Average read length	Number of OTUs	Chao1 index	Shannon index	Simpson index	Observed species	Good’s coverage^2^
Fresh	783	1240	41	49.7	3.203	0.78	40.8	0.96
C-14	220	1242	10	11.5	2.57	0.78	8.5	0.96
C-30	124	1248	14	92	1.64	0.40	5.4	0.77
C-60	382	1249	21	27	2.33	0.59	19.8	0.95
C-90	636	1246	24	35	1.96	0.51	23.6	0.96
LB-14	726	1252	42	96.2	3.22	0.81	41.4	0.91
LB-30	969	1251	57	117.5	3.15	0.78	50.1	0.92
LB-60	1052	1247	45	138.5	1.16	0.24	28.5	0.94
LB-90	1418	1245	71	106.1	2.19	0.47	42.3	0.95
LP-14	1564	1246	45	68.0	0.93	0.19	21.7	0.98
LP-30	1431	1252	68	135.6	1.55	0.31	36.8	0.95
LP-60	1052	1247	61	147.1	2.12	0.49	44.3	0.93
LP-90	601	1247	36	91.2	2.68	0.69	33.3	0.92

**Figure 2:**
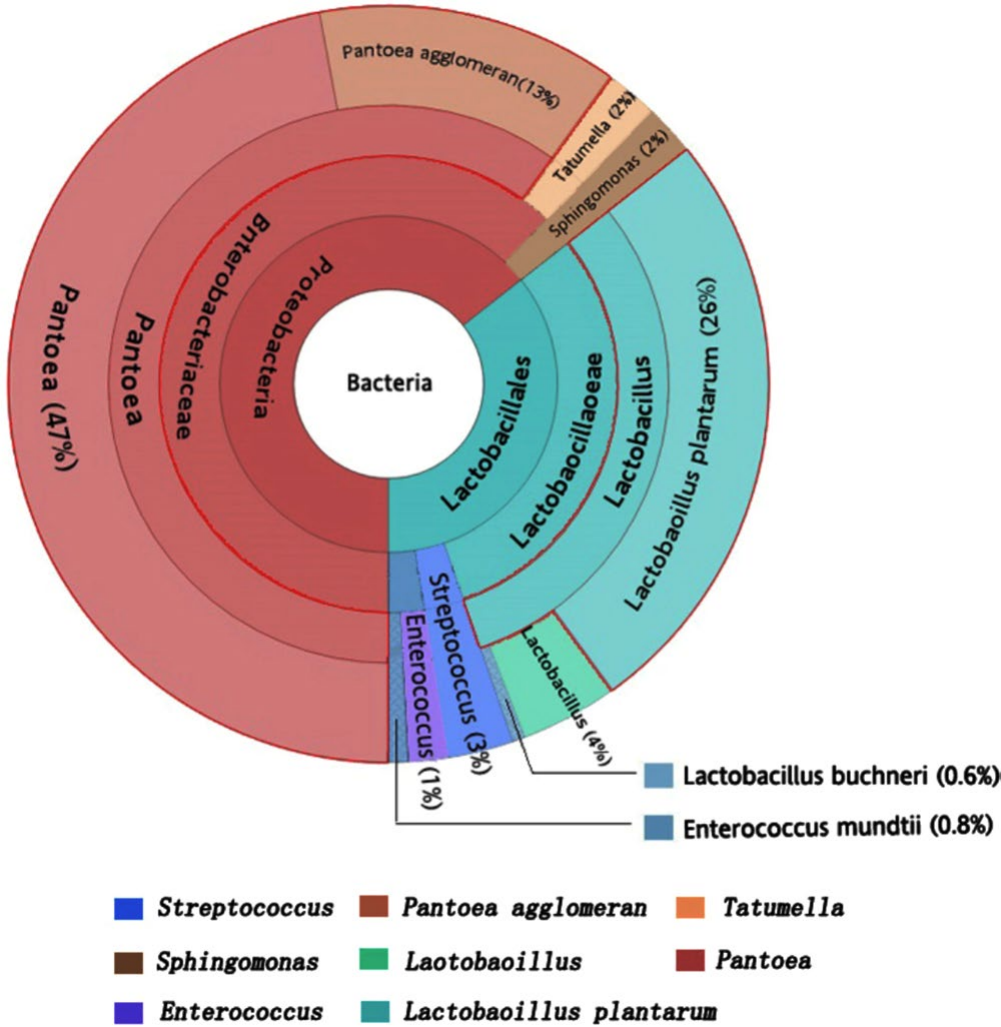
Epiphytic bacteria composition in fresh alfalfa before ensiling.

**Figure 3:**
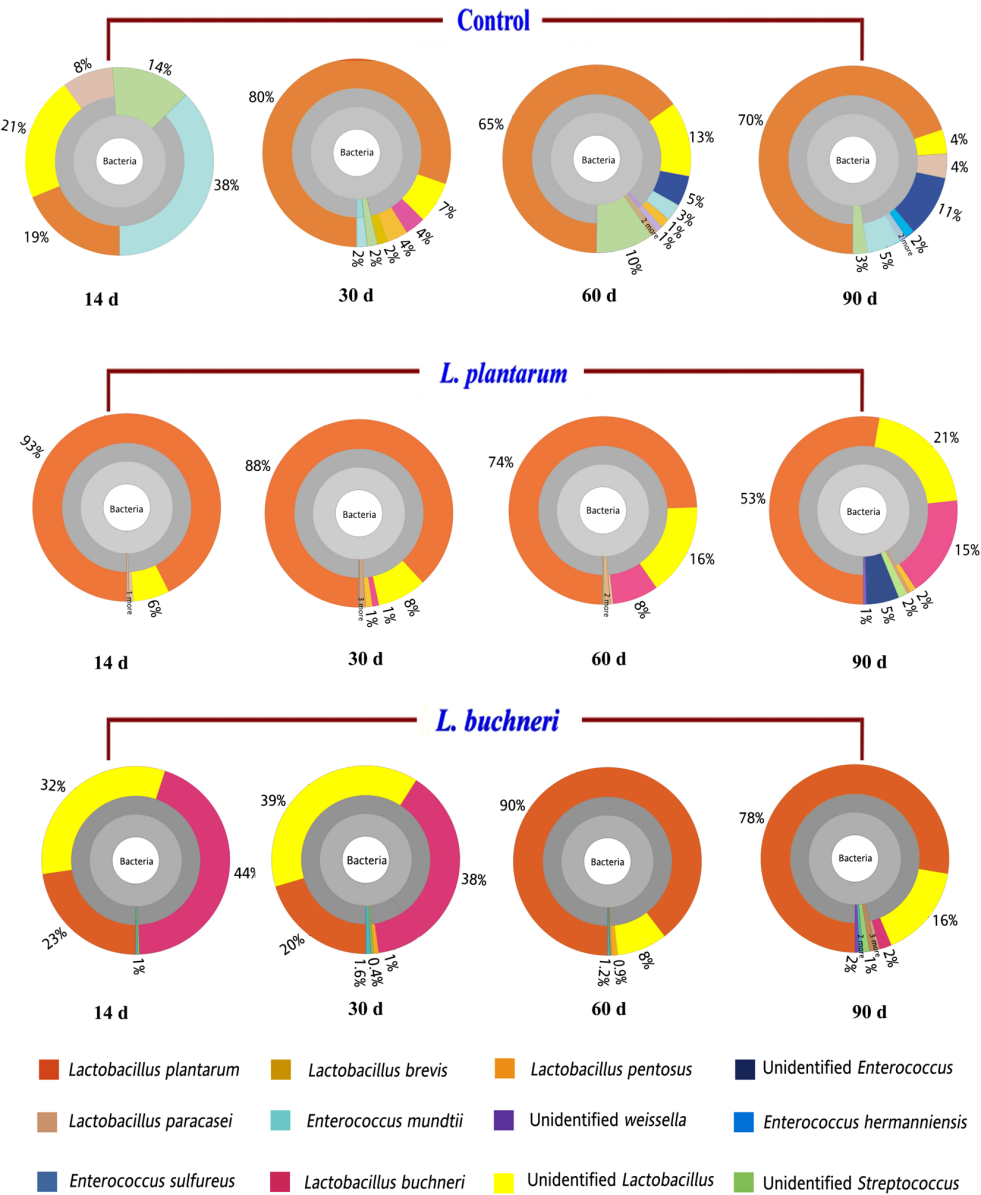
Comparison of taxonomic profiles of ensiling microbial communities of control, *L*. *plantarum*-inoculated and *L*. *buchneri*-inoculated silages after 14, 30, 60, and 90 days of fermentation. Metagenomic reads were taxonomically assigned based on the Ribosomal Database Project II database with a minimum bootstrap threshold of 80% and visualization by Krona.

The epiphytic microflora of forage before ensiling comprised *Pantoea*, *Lactobacillus*, *Enterococcus*, *Streptococcus*, *Sphingomonas*, *Tatumella*, and others, in which *Pantoea* was the predominant bacteria showing 57% relative abundance followed by *L. plantarum* (27%; Fig. 2). After ensiling, the observed microflora included the genera *Lactobacillus*, *Enterococcus*, *Streptococcus*, and *Weissella*, and completely different microbial community dynamics were observed in the control, *L. buchneri*-inoculated, and *L. plantarum*-inoculated silages (Fig. 3). After 14 days of fermentation, the dominant LAB in control silages was *Enterococcus mundtii* (38%), followed by unidentified *Lactobacillus* (21%), *L. plantarum* (19%), unidentified *Streptococcus* (14%), and *Lactobacillus paracasei* (8%). As fermentation time of the control silages increased, the dominant microbial species was *L. plantarum*, showing relative abundance values of 80%, 65%, and 70% in 30-, 60-, and 90-day silages, respectively. The bacterial microbiota in *L. buchneri*-inoculated silages ensiled for 14 and 30 days was predominated by *L. buchneri* (38–44%), unidentified *Lactobacillus* (32–39%), and *L. plantarum* (20–23%), whereas 90% and 78% of the microbiota was *L. plantarum* after 60 and 90 days of fermentation, respectively. No *L. buchneri* was observed in 60-day silages. Inoculation of *L. plantarum* markedly promoted the growth of *L. plantarum* in ensiled alfalfa as showed by its predominant occupation throughout the fermentation process. However, the proportion of *L. plantarum* in the total microbiota decreased from 93% to 53% and proportion of unidentified *Lactobacillus* increased when the fermentation time increased from 14 to 90 days. The *L. buchneri* was not detectable in *L. plantarum*-inoculated silages fermented for 14 days, but the species appeared in 30-day silages (1%) and then propagated over increased fermentation time until its relative abundance reached 17% in 90-day silages.

**Figure 4:**
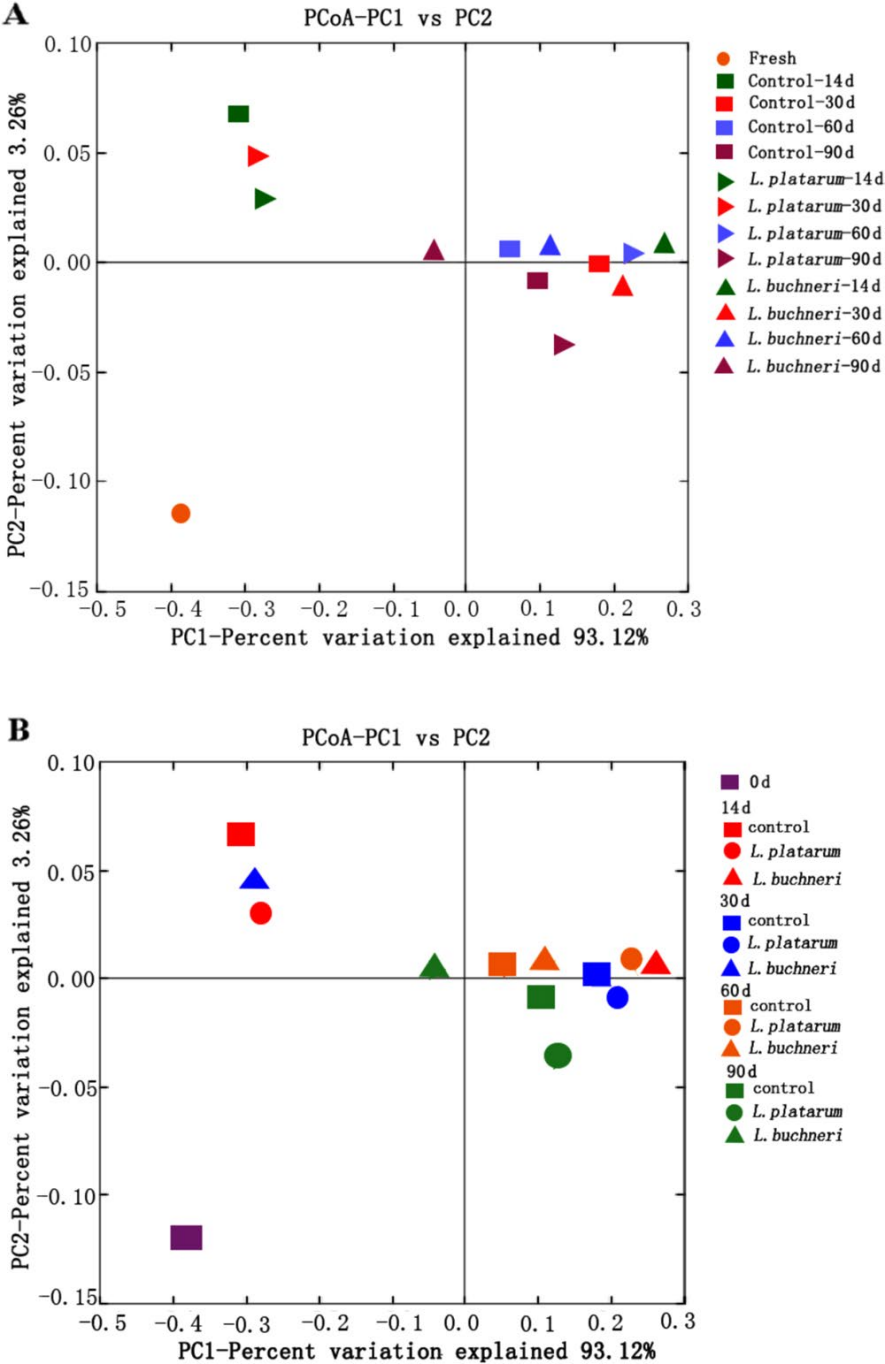
UniFrac weighted principle coordinate analysis scores plot based on PC1 and PC2. (**A**) Treatment (different shapes represent treatments, and one shape with different colors represent ensiling days); (**B**) Ensiling day (different colors represent ensiling days, and one color with different shapes represent treatments).

Principal coordinate analysis based on the weighted UniFrac distance showed that distinct clusters were identified between the fresh forage and the ensiled forages with different inoculant treatments and fermentation days (Fig. 4A and B). There was less variation in microbial diversity in *L. buchneri*-inoculated silages for the four different fermentation days compared to in the control and *L. plantarum*-inoculated silages. However, microbial diversity in the control and *L. plantarum*-inoculated silages fermented for 14 days as well as in the *L. buchneri*-inoculated silages fermented for 30 days were clearly separated from that in the remaining control, *L. plantarum*-inoculated and *L. buchneri-*inoculated silages.

## Discussion

It is difficult to generate high-quality silage using alfalfa because of its high buffer capacity and low fermentable carbohydrate concentration^16^. Therefore, inoculants are commonly used to rapidly decrease silage pH and ensure the fermentation quality of ensiled alfalfa^15,17^. Fermentation parameters, such as pH and lactic and acetic acid concentrations of the ensiled alfalfa, suggest that high quality-silages were prepared in our mini-silos. All silages showed a pH value of approximately 5, which is similar to the results of previous studies of alfalfa silage^4,16^. The concentrations of lactic and acetic acid in silages were within reported concentration ranges^4,15–17^. The low amount of ammonia present in the silos indicates minimal clostridia fermentation and amino acid deamination.

In addition to metabolites that are commonly detected in silage, metabolomic profiling analysis revealed metabolites that had not been previously detected in silage. In the present study, a total of 280 substances were detected in the ensiled alfalfa, and 102 different metabolites were identified. Inoculation of the homofermenter *L. plantarum* or heterofermenter *L. buchneri* clearly modulated the metabolite composition pattern of the ensiled forage. Over the past two decades, studies of the heterofermenter *L. buchneri* have mainly focused on its fermentative metabolites acetic acid and 1,2-propanediol, which increase the stability of silage against deterioration by yeasts and molds when exposed to air^12–18^. Our results showed that inoculation of *L. buchneri* resulted in an up-accumulation in the concentrations of 4-aminobutyric acid, some free amino acids (threonine, tyrosine, valine, ornithine, lysine, beta-alanine, aspartic acid), and polyols (2,3-butandiol, glycerol, arabitol, erythritol, mannitol, threitol) in ensiled alfalfa. Among the up-accumulated free amino acids, valine and aspartic acid were present at considerably high relative concentrations, suggesting that *L. buchneri* can produce large amounts of these two free amino acids during fermentation. 4-Aminobutyric acid is a non-protein amino acid in animals; it functions as a major inhibitory neurotransmitter and can decrease blood pressure^19^. The detected polyols are well-known sweetening or flavor agents. In addition, *L. buchneri* inoculation also caused remarkable accumulation of cadaverine in silages, which agrees with the results of previous studies^20^. High accumulation of biogenic amines produced by some strains of *L. buchneri* may lower the palatability or eating rate of the silage^21^. However, silages inoculated with *L. plantarum* showed a significant down-accumulation in cadaverine. Compared with *L. plantarum* inoculated-silages, the up-accumulated polyols and free amino acids in *L. buchneri* inoculated-silages maybe due to the unique metabolic pathways of sugar fermentation and amino acid biosynthesis performed by *L. buchneri* or the unidentified *Enterococcus* consumed polyols and free amino acids in *L. plantarum* inoculated-silages.

Based on the above results, some functional ingredients and flavoring agents in ensiled alfalfa were detected after inoculation with *L. plantarum* or *L. buchneri*, suggesting that metabolomic profiling analysis is a powerful tool for comprehensively evaluating the fermentative, nutritive, and functional profiles of ensiled forages for animals. In the present study, however, we did not optimize the temperature programming for metabolite analysis using GC-MS in ensiled alfalfa samples. Therefore, developing an optimal temperature program suitable for analyzing metabolites in the fermentative system for ensiled forages is necessary to reveal all metabolites present.

Ensiling without inoculation or starter cultures is a spontaneous fermentative process that depends on the epiphytic microbial composition and natural occurrence of epiphytic LAB. Before ensiling, the most dominant epiphytic bacteria in alfalfa forage were members of the genus *Pantoea* and species *Pantoea agglomerans* and *L. plantarum*. However, a previous report showed that the genera *Erwinia*, *Escherichia*, *Pseudomonas*, *Pantoea*, and *Enterobacter* were the predominant microbial flora in the alfalfa phyllosphere, and the authors suggested that the colonization of plant surfaces by bacteria depends on many factors including plant species, climate, geographical location, and type of fertilizer used^4^. The bacterial flora in control silages showed a remarkable shift during the ensiling process, from predominantly *E. mundtii* (38%), *L. plantarum* (19%), unidentified *Lactobacillus* (21%) and *Streptcoccus* (14%) in 14-day silages to mostly *L. plantarum* (70%) followed by unidentified *Enterococcus* (11%) in 90-day silages. In contrast to previous results, which showed that *L. buchneri* was the most abundant LAB in alfalfa silage fermented for 60 days^4^, our results indicated that during ensiling from 30 to 90 days, *L. plantarum* was the most abundant LAB with a relatively abundance of 65–80%. Species of *L. buchneri*, *L. pentosus*, and *L. brevis* were present in 30- and 60-day silages but undetectable in 90-day silages.

Two types of inoculants, homofermentative and heterofermentative LAB, have been widely used for silage preservation. Homofermentative LAB strains are used because they produce 2 moles of lactic acid for each mole of glucose fermented and can quickly lower the pH of the ensiled material. Heterofermentative LAB produce high concentrations of acetic acid in addition to lactic acid and other products, thus improving the aerobic stability of ensiled forage by inhibiting fungi growth when silage is exposed to air^22^. Thus, the two types of inoculants use different approaches to direct fermentation during the ensiling process, and understanding the interactions between these inoculants and other microorganisms in silage fermentation may help to improve the process^5,15^. As expected, inoculation with *L. plantarum* before ensiling of alfalfa resulted in its predominant occupation among the microbial flora of ensiled alfalfa from 14 to 90 days of ensiling, as reported previously^23^, but a constant down-regulation in its relative abundance was observed from 93% in 14-day silages to 53% in 90-day silages. The up-regulation in relative abundance of strain *L. buchneri* in *L. plantarum*-inoculated silages from 30 days to the end of the ensiling process suggests that *L. buchneri* plays a role in the late stage of fermentation and is tolerant to the acid environment. Interestingly, *L. buchneri* appeared in 30-day control silages but was not detectable in 60- or 90-day silages because of its weak competition with other microbes. These results indicate that inoculation with *L. plantarum* increases the competitiveness of *L. buchneri* to colonize at late fermentation stages in ensiled alfalfa. In *L. plantarum*-inoculated silages, *L. plantarum* accounted for as much as 93% of the total microbes in 14-day silages, whereas inoculation with *L. buchneri* resulted in a relative abundance of 44% in 14-day silages. Therefore, *L. buchneri* showed less competitive colonizing behavior at earlier fermentation stage than *L. plantarum*. Moreover, a dramatic shift in microbial composition occurred in *L. buchneri*-inoculated silages fermented for 60 days, and the high relative abundance of *L. plantarum* (90%) indicating that *L. plantarum* was more competitive in colonization than *L. buchneri* at late stages of fermentation. No *L. buchneri* was detected in 60-day silages and only a small amount (2%) of this strain was observed in 90-day silages. According to a previous study, the strain of *L. buchneri* was detectable in Italian ryegrass silage inoculated with *L. buchneri* throughout its ensiling process from 14 to 120 days^24^. The inconsistent results may be due to different grass species and epiphytic bacterial compositions of fresh material used before ensiling^4^. In the present study, we did not investigate the associability between the dynamics of the silage microbiota and metabolome. However, our results provide a foundation for future silage research on silage metabolome and microbiome. A better understanding of the relationship between the silage microbiome and metabolome as well as how inoculants drives this relationship will be much helpful for manipulating the silage fermentation process and quality.

In summary, we found a total of 102 annotated metabolites in alfalfa silages, and completely different composition patterns of metabolites were observed in the control, *L. plantarum*-inoculated, and *L. buchneri-*inoculated silages. Inoculation with *L. buchneri* resulted in remarkable up-accumulations in concentrations of 4-aminobutyric acid, some free amino acids, and polyols, whereas considerable down-accumulations in cadaverine and succinic acid were observed in *L. plantarum*-inoculated silages. Inoculations with homofermentative *L. plantarum* or heterofermentative *L. buchneri* altered the microbial composition (identified at the species level) dynamics of ensiled forage in very different manners compared to in control silages. These findings show that metabolomic profiling analysis can be used to evaluate ensiled forages not only in terms of fermentation quality, but also based on nutritional and functional metabolites that are beneficial to animal health and welfare. Additionally, by using the PacBio SMRT method, the microbial composition and its succession during the ensiling process were revealed at the species level, providing valuable biological information regarding the complete microbial community in the ensiling system. Our results improve the understanding of the microbial processes underlying silage formation and may be helpful for developing target-based regulation methods to produce high-quality silage.

## Methods

### Ensiling of grass samples

Alfalfa (*Medicago sativa* L.) was mowed in the late bud to early bloom stage and wilted to a dry matter (DM) content of 421 ± 7.3 g/kg fresh weight (FW). The Crude protein (CP), neutral detergent fiber (NDF), acid detergent fiber (ADF), water-soluble carbohydrate (WSC) and ammonia nitrogen (NH_3_-N) in the fresh alfalfa were 184 ± 1.6 g/kg DM, 495 ± 6.4 g/kg DM, 288 ± 4.7 g/kg DM, 28.4 ± 0.67 g/kg DM and 4.13 ± 0.05 g/kg total N, respectively. The lactic acid bacteria, yeasts and molds in the fresh alfalfa were 6.51 ± 0.04, 6.38 ± 0.02 and 6.36 ± 0.01 log_10_ cfu/g, respectively. The harvest forage was chopped into 1–2 cm pieces using a paper cutter. Vacuumized sixteen silos (vacuum-sealing polyethylene plastic bags packed with approximately 300 g of fresh forage) were individually prepared for each of the following treatments: (a) untreated (control), (b) *L. plantarum*, and (c) *L. buchneri*. The application rate of each inoculant into the fresh forage was 1 × 10^6^ cfu/g. To apply the inoculants to the chopped alfalfa forage, *L. plantarum* or *L. buchneri* was dissolved in 10 mL distilled water and mixed thoroughly with the forages after uniform spraying onto the piles prepared for each treatment. To the untreated forage sample, the same amount of distilled water was applied. The silos were then stored at ambient temperature (22–25 °C) in dark condition and sampled at 18:00 on days 14, 30, 60, and 90 for later analysis.

### Chemical analysis and microbial composition enumeration

The 90-d silos were opened and a portion of silage was immediately frozen (−20 °C) in sealed plastic bags until further chemical analysis. Initial fresh forage samples were taken before the samples were ensiled. A 20 g FW sample from each bag was placed in a juice extractor, diluted with 180 mL distilled water, squeezed for 30 s at a high speed, and filtered through four layers of medical gauze. The filtrate was divided into two parts. The pH was measured immediately; next, one part of the filtrate was acidulated with 7.14 M H_2_SO_4_ and filtered with a 0.45-μm dialyzer. Lactic acid, acetic acid, propionic acid, and butyric acid were analyzed by high-performance liquid chromatography (KC-811 column, Shodex; Shimadzu, Kyoto, Japan; oven temperature, 50 °C; flow rate, 1 mL/min; SPD, 210 nm). One milliliter of 250 g/L (w/v) trichloroacetic acid was added to 4 mL of the second part of the filtrate from each bag. This solution was incubated overnight at 4 °C to precipitate the protein and then centrifuged at 4 °C, 18,000 × *g* for 15 min. Next, the supernatant fluid was analyzed for NH_3_-N as described by Broderick and Kang^25^ and for WSC as described by Thomas^26^.

The DM content of the remaining ensiled forage from each silo was measured by drying the samples in a forced-air oven at 65 °C for 72 h. Dried samples were ground with a mill (1-mm screen). Ground samples were analyzed for Kjeldahl N^27^. The CP was calculated as Kjeldahl N × 6.25. The contents of NDF and ADF were determined as described by Van Soest *et al*.^28^ using an Ankom 200 fiber analyzer (Ankom Technology, Fairport, NY, USA). During analysis, heat-stable alpha amylase and sodium sulfite were added. The NDF and ADF were expressed with residual ash.

### Metabolite profiling analysis

For silage extraction, a portion of the ensiled alfalfa from 90-day silos was sampled from three silos of each treatment. Ensiled alfalfa leaves were separated, and 5 g leaves were suspended gently in 50 mL methanol in a shaker at 100 rpm for 2 min. The suspension fluid was centrifuged for 10 min at 11,000 × *g*. The supernatants were then subjected to derivatization. Briefly, the supernatant samples (each sample 500 µL) were blow-dried by moderate nitrogen, followed by addition of 60 µL of 15 mg mL^−1^ methoxyamine pyridine solution, vortex mixing for 30 s, and a reaction overnight of 16 h at room temperature. Finally, 60 µL BSTFA reagent (containing 1% TMCS) was added to the mixture and reacted for 60 min at room temperature of 20–23 °C. The final reacted samples were analyzed for metabolite composition with a 7890 A/5975 C GC-MS apparatus (Agilent Technologies, Santa Clara, CA, USA) equipped with a fused silica capillary column HP-5MS (5% phenyl methyl silox: 30 m × 250 µm i.d., 0.25 µm; Agilent J&W Scientific, Folsom, CA, USA). Samples (1 µL) were injected in split mode (split ratio 20:1) with helium used as the carrier gas at a flow rate of 1.0 mL min^−1^. The temperatures of the injector, ion source, and connector were kept at 280 °C, 250 °C, and 150 °C, respectively. The oven temperature was programmed as follows: initial temperature of 40 °C maintained for 5 min, increased to 300 °C at 10 °C per min, and then maintained for 5 min. Mass spectrometry conducted using a full-scan method from 35 to 780 (m/z). The raw signals exacting, data baselines filtering, peak identification, and integration were performed using XCMS software (www.bioconductor.org/) as described previously^29^. The total mass of the signal integration area was normalized to 10,000 for each sample. The normalized data were then imported into the Simca-P software (version 11.5) to detect differentially expressed metabolites. The NIST (http://www.nist.gov/index.html) and KEGG (http://www.genome.jp/kegg/) commercial databases were used to search for metabolites.

### Microbial composition SMRT analysis

Fresh alfalfa and ensiled alfalfa from the control and *L. plantarum*- and *L. buchneri*-inoculated groups fermented for 14, 30, 60, and 90 days were sampled for total bacteria DNA extraction. After extracting DNA from samples of the four replicates at each fermentation day, the extracted DNA was equally mixed into one sample for later analysis. Therefore, a total of 13 samples, including 1 fresh material sample (before ensiling) and 12 samples from control, *L. plantarum*-inoculated and *L. buchneri*-inoculated groups, were collected. Genomic DNA was extracted using a DNA isolation kit (Tiangen, DP302-02, Tiangen, China) and the quality of extracted DNA samples was evaluated by 1% agarose gel electrophoresis and spectrophotometry, according to the manufacturer’s protocol. All samples were purified through a QIAamp DNA Stool kit column (Qiagen, Hilden, Germany) and stored at −20 °C until further analysis. PCR amplification of the full-length 16S rRNA gene for SMRT sequencing was carried out using the forward primer 27 F (5′-GAGAGTTTGATCCTGGCTCAG-3′) and reverse primer 1541 R (5′-AAGGAGGTGATCCAGCCGCA-3′). Both primers contained a set of 16-nucleotide barcodes. The PCR program was 95 °C for 2 min, 30 cycles of 95 °C for 60 s, 60 °C for 45 s, and 72 °C for 60 s, with a final extension of 72 °C for 7 min^6^. Sequencing of the amplicons was performed on a PacBio RS II instrument (Pacific Biosciences, Menlo Park, CA, USA) using P6-C4 chemistry, as described previously^30^.

Raw data were processed with the protocol RS_Readsofinsert.1 of SMRT Portal version 2.7 (Pacific Biosciences). High-quality sequences were extracted using the Quantitative Insights Into Microbial Ecology (QIIME) package (version 1.7) and aligned by PyNAST^31^ and UCLUST^32^ under 100% clustering of sequence identity to obtain representative sequences. The unique sequence set was classified into OTUs under 98.6% threshold identity using UCLUST^33^. The potential chimerical sequences in the representative set of OTUs were removed by ChimeraSlayer^34^. Subsequently, the taxonomy of each OTU representative sequence was assigned using the Ribosomal Database Project II database classified at a minimum bootstrap threshold of 80%^35^. Taxonomic assignments were visualized using Krona^36^. After constructing a *de novo* taxonomic tree with a chimera-checked representative OTU set in Fast Tree, alpha diversity index (Shanno-Wiener, Simpson’s diversity, Chao1, and rarefaction) was calculated using QIIME software. The UniFrac distance was calculated based on the phylogenetic tree for principal coordinate analysis^37^. The graph presentations were generated using R package version 3.1.2 and Original software version 8.5. The sequence data reported in this study have been deposited in the MG-RAST database (Accession No. 4794169.3-4794181.3).

### Statistical analysis

Data on silage fermentation and biochemical compositions were subjected to the one-way analysis of variance using SAS software (SAS version 9.0, SAS Institute, Inc. Cary, NC, USA). All metabolite data were normalized using Simca-P software (version 11.5, http://www.umetrics.com/simca) before hierarchical cluster analysis and principal component analysis. Significant differences in metabolites among treatment groups were tested by one-way analysis of variance. Quantitative normalization within replicates was transformed by a logarithmic base of 2 and MetaboAnalyst online analysis software (www.metaboanalyst.ca/) was used to build a heatmap diagram. *P*-values below 0.05 between sample groups were considered significant.

## Electronic supplementary material

Supplementary tables and figures

Dataset 1
