# Supplementary material for: Profiling of metabolome and bacterial community dynamics in ensiled Medicago sativa inoculated without or with Lactobacillus plantarum or Lactobacillus buchneri
Source: Sci Rep. 2018 Jan 10;8:357. doi: 10.1038/s41598-017-18348-0 (PMC5762819; doi:10.1038/s41598-017-18348-0)
Supplement: Supplementary file 1 — Supplementary tables and figures [file 41598_2017_18348_MOESM1_ESM.doc]

**Profiling of** **metabolome and bacterial community dynamics in ensiled *Medicago sativa* inoculated without or with *Lactobacillus plantarum* or *Lactobacillus buchneri***

**X. S. Guo^1,2*^, W. C. Ke^1,2^, W.R. Ding****^1,2^, L. M. Ding^1^, D. M. Xu^1,2^, W. W. Wang^1^, P. Zhang ^1,2^, F.Y. Yang^3^**

^1^State Key Laboratory of Grassland and Agro-ecosystems, School of Life Sciences, Lanzhou University, Lanzhou 730000, PR China

^2^Probiotics and Biological Feed Research Center, Lanzhou University, Lanzhou 730000, PR China

^3^Institute of Grassland Science, College of Animal Science and Technology, China Agricultural University, Beijing 100193, PR China

*Corresponding authors: Dr. Xusheng Guo; Dr. Fuyu Yang.

Tel.: +86 931 8915650

Fax: +86 931 8915650

E-mail addresses: [guoxsh07@lzu.edu.cn](mailto:guoxsh07@lzu.edu.cn) (X.S. Guo); yfuyu@126.com

**Table S1. The contribution of 102 indentified metabolites to the first principal component (PC1) and the second principal component (PC2)**

| **Metabolites** | **PC1** | **PC2** |
| --- | --- | --- |
| Homoserine | 0.086038 | 0.102907 |
| N-Methoxy-Amine | 0.120808 | 0.029165 |
| Ethanolamine | 0.07689 | 0.107426 |
| Ethylene glycol | -0.06762 | -0.01098 |
| 2,3-Butandiol | -0.1639 | -0.00871 |
| 2,3-Butanediol | 0.133752 | -0.12462 |
| Lactic acid | 0.050902 | 0.175474 |
| Hexanoic acid | 0.013505 | 0.178889 |
| Alanine | -0.00084 | -0.01847 |
| alpha-Hydroxyisobutyric acid | 0.124192 | -0.14156 |
| 2-Aminobutyric acid | 0.10366 | -0.16079 |
| Malonic acid | -0.09046 | 0.177111 |
| Valine | -0.12934 | -0.0368 |
| Phenol | -0.07233 | 0.103823 |
| 4-Hydroxybutanoic acid | -0.12505 | 0.012843 |
| Benzoic acid | -0.09986 | 0.106804 |
| Leucine | 0.140709 | 0.035791 |
| Glycerol | -0.13581 | -0.07967 |
| Isoleucine | 0.007296 | 0.008276 |
| Proline | 0.006398 | 0.03664 |
| Glycine | -0.13493 | 0.013233 |
| Succinic acid | 0.082057 | -0.1687 |
| Glyceric acid | 0.08928 | -0.16251 |
| Uracil | -0.1176 | -0.07585 |
| Serine | -0.08532 | 0.082053 |
| Pipecolic acid | -0.10665 | 0.003213 |
| Threonine | -0.14046 | 0.068755 |
| Thymine | -0.09877 | -0.073 |
| beta-Alanine | -0.14084 | -0.03595 |
| Putrescine | -0.03512 | 0.119529 |
| Ketomalonic acid | -0.04582 | 0.204034 |
| Aminomalonic acid | -0.13554 | 0.063449 |
| Malic acid | -0.00579 | -0.08741 |
| Threitol | -0.15356 | -0.02078 |
| Salicylic acid | -0.1166 | 0.048566 |
| Erythritol | -0.14944 | -0.07499 |
| Aspartic acid | -0.13735 | 0.102786 |
| 4-Aminobutyric acid | -0.15151 | -0.0046 |
| Erythronic acid | -0.0138 | -0.03015 |
| Phenethylamine | 0.106188 | -0.16657 |
| Threonic acid | -0.01623 | -0.0239 |
| Hydroxyglutaric acid | 0.087646 | -0.06288 |
| Phenyllactic acid | 0.118002 | 0.124329 |
| Xylulose | -0.07922 | -0.01479 |
| 3-Hydroxy-3-methylglutaric acid | -0.06265 | -0.05023 |
| 2-Deoxy-pentitol | -0.13236 | -0.09461 |
| 1-Methyl-beta-D-galactopyranoside | -0.0655 | -0.14989 |
| Glutamic acid | 0.148831 | -0.09136 |
| Phenylalanine | -0.14098 | 0.025293 |
| Xylonic acid-lactone | 0.003032 | 0.168378 |
| Pentitol | -0.12453 | -0.0866 |
| Xylose | -0.05266 | -0.09266 |
| Dodecyl acrylate | 0.063236 | 0.089251 |
| Arabitol | -0.15496 | -0.05582 |
| 2-Amino-Adipinic Acid | -0.11189 | 0.08042 |
| Xylitol | -0.10725 | -0.08163 |
| Fucose | -0.09734 | -0.06154 |
| Eicosanol | -0.05615 | 0.131316 |
| 4-Hydroxyphenylpropionic Acid | -0.01966 | 0.203638 |
| Isovanillic Acid | -0.11626 | 0.014358 |
| 2,6-dihydroxy-benzoic acid | -0.00594 | 0.126338 |
| Ribonic acid | -0.0806 | -0.02491 |
| Ornithine | -0.12463 | 0.005832 |
| Neophytadiene | 0.095546 | 0.035518 |
| Cadaverine | 0.073195 | -0.18549 |
| Tetradecanoic acid | -0.11319 | -0.06703 |
| Pinitol | -0.09407 | 0.049589 |
| Adenine | -0.10978 | 0.154835 |
| Viburnitol | -0.05023 | -0.08323 |
| Vanillylpropionic acid | -0.07864 | 0.15368 |
| Allantoin | -0.10938 | 0.103007 |
| Galactose | 0.116753 | -0.06508 |
| Tyramine | -0.08365 | -0.11706 |
| Lysine | -0.13133 | 0.057939 |
| p-Coumaric acid | 0.079078 | -0.07316 |
| Tyrosine | -0.13009 | 0.100183 |
| Mannitol | -0.14102 | -0.07017 |
| Glucuronic acid | -0.10842 | 0.106675 |
| Ononitol | -0.09359 | -0.02234 |
| Ethyl hexadecanoate | -0.12982 | -0.08454 |
| Hexadecanoic acid | -0.12321 | -0.02455 |
| Ethyl linoleate | -0.00543 | -0.12293 |
| trans-Ferulic acid | -0.06257 | -0.14294 |
| Methyl linolenate | -0.07114 | 0.002045 |
| Inositol | -0.09438 | 0.105511 |
| Heptadecanoic acid | -0.11111 | -0.06878 |
| Sedoheptulose | -0.10684 | 0.03626 |
| Linoleic acid ethyl ester | -0.13238 | -0.10537 |
| Ethyl linolenate | -0.13742 | -0.09751 |
| Phytol | -0.11754 | -0.03646 |
| 9,12-(Z,Z)-Octadecadienoic acid | -0.0386 | -0.13449 |
| Linolenic acid | -0.05777 | -0.11678 |
| Tryptophan | -0.02545 | -0.13941 |
| Stearic acid | -0.04311 | -0.05166 |
| Pseudouridine | -0.12459 | 0.035687 |
| D-Glucuronic acid | -0.06567 | -0.05513 |
| Eicosanoic acid | -0.00373 | -0.10003 |
| Monopalmitin | 0.03276 | 0.138619 |
| Sucrose | 0.082118 | -0.06347 |
| Melezitose | -0.03552 | -0.12964 |
| Maltose | -0.01573 | -0.16175 |
| Cellobiose | -0.07 | 0.091915 |

**
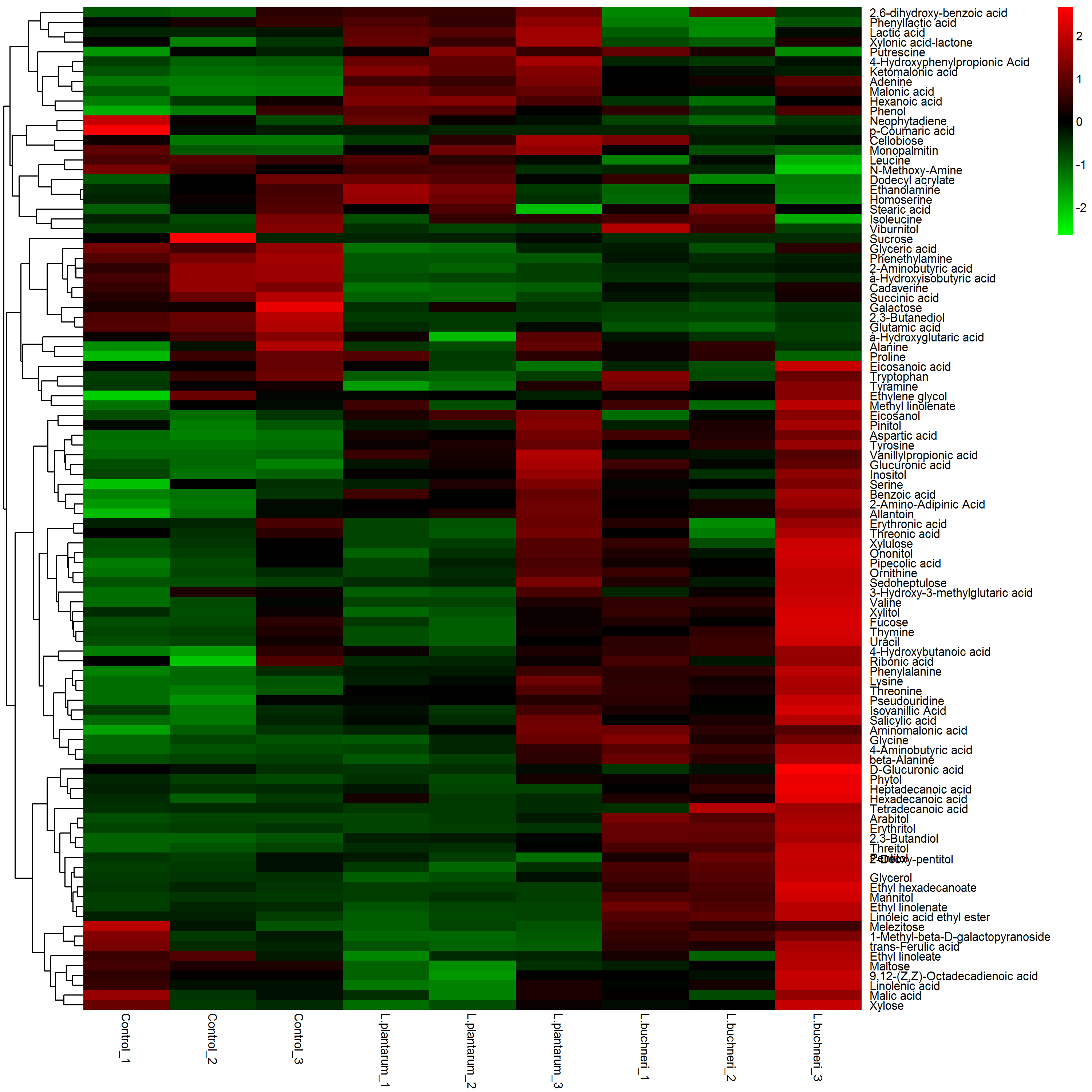
**

**Figure S1. Heatmap analysis combined with hierarchical cluster analysis (HCA) of 102 metabolites in the control silage and silages inoculated by *Lactobacillus plantarum* or *Lactobacillus buchneri*.**


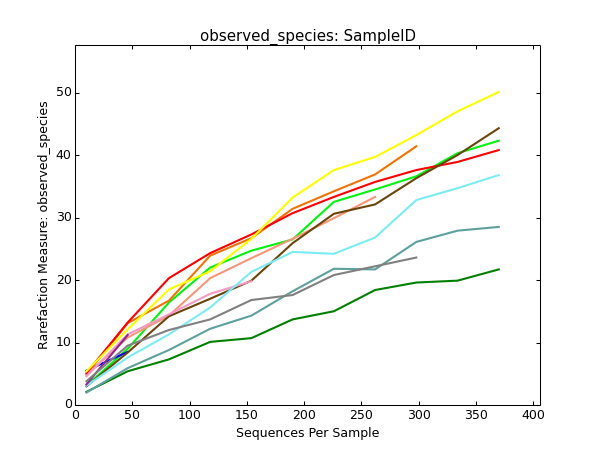

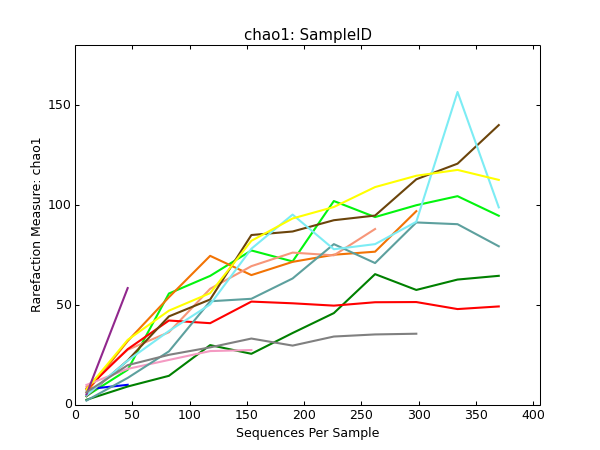


**Figure S2. Shannon diversity index curves showing the diversity of taxa present in silages**
