# Supplementary material for: Profiling of metabolome and bacterial community dynamics in ensiled Medicago sativa inoculated without or with Lactobacillus plantarum or Lactobacillus buchneri
Source: Sci Rep. 2018 Jan 10;8:357. doi: 10.1038/s41598-017-18348-0 (PMC5762819; doi:10.1038/s41598-017-18348-0)
Supplement: Supplementary file 2 — Dataset 1 [file 41598_2017_18348_MOESM2_ESM.doc]

**Profiling of** **metabolome and bacterial community dynamics in ensiled *Medicago sativa* inoculated without or with *Lactobacillus plantarum* or *Lactobacillus buchneri***

**X. S. Guo^1,2*^, W. C. Ke^1,2^, W.R. Ding****^1,2^, L. M. Ding^1^, D. M. Xu^1,2^, W. W. Wang^1^, P. Zhang ^1,2^, F.Y. Yang^3^**

^1^State Key Laboratory of Grassland and Agro-ecosystems, School of Life Sciences, Lanzhou University, Lanzhou 730000, PR China

^2^Probiotics and Biological Feed Research Center, Lanzhou University, Lanzhou 730000, PR China

^3^Institute of Grassland Science, College of Animal Science and Technology, China Agricultural University, Beijing 100193, PR China

*Corresponding authors: Dr. Xusheng Guo; Dr. Fuyu Yang.

Tel.: +86 931 8915650

Fax: +86 931 8915650

E-mail addresses: [guoxsh07@lzu.edu.cn](mailto:guoxsh07@lzu.edu.cn) (X.S. Guo); yfuyu@126.com

**File S1.** The relative concentration of 102 indentified metabolites in the control, *Lactobacillus plantarum*- and *Lactobacillus buchneri*-inoculated silages with triplicate for each treatment^1^

| Annotation | Control_1 | Control_2 | Control_3 | *L.plantarum*_1 | *L.plantarum*_2 | *L.plantarum*_3 | *L.buchneri*_1 | *L.buchneri*_2 | *L.buchneri*_3 |
| --- | --- | --- | --- | --- | --- | --- | --- | --- | --- |
| 1-Methyl-beta-D-galactopyranoside | 3.3578442 | 1.3934869 | 1.7245094 | 0.943778388 | 0.935994673 | 1.10978887 | 2.587079784 | 2.784714863 | 3.316249062 |
| 2,3-Butandiol | 82.347374 | 81.145644 | 96.293318 | 157.7607256 | 164.4231618 | 190.7483065 | 316.3268444 | 309.8945793 | 394.9452366 |
| 2,3-Butanediol | 70.82838 | 78.682703 | 102.74058 | 28.07822305 | 27.59903683 | 28.73567313 | 24.70863581 | 24.44893092 | 31.62577421 |
| 2,6-dihydroxy-benzoic acid | 4.977356 | 4.4071847 | 10.629696 | 11.28625802 | 10.93752393 | 13.70372769 | 2.93742775 | 13.51307361 | 6.126253479 |
| 2-Amino-Adipinic Acid | 0 | 0.02663 | 0.1205307 | 0.135104646 | 0.131900365 | 0.230533189 | 0.13194541 | 0.153288732 | 0.264890433 |
| 2-Aminobutyric acid | 45.329406 | 68.272871 | 70.90865 | 11.35488021 | 8.791730498 | 17.47709765 | 22.70690945 | 24.92816613 | 27.7924564 |
| 2-Deoxy-pentitol | 3.6176037 | 3.6718365 | 4.3362449 | 3.648711575 | 2.881826093 | 3.835316193 | 5.843165104 | 6.059297598 | 7.696096811 |
| 3-Hydroxy-3-methylglutaric acid | 1.226701 | 1.7416079 | 1.6732155 | 1.272978277 | 1.322974008 | 1.896516197 | 1.481358845 | 1.672321363 | 2.334266958 |
| 4-Aminobutyric acid | 98.097092 | 114.39388 | 124.74795 | 130.7139133 | 153.4442974 | 223.2904016 | 257.486631 | 234.9226562 | 324.1801957 |
| 4-Hydroxybutanoic acid | 0.2851626 | 0.1139307 | 1.0973807 | 0.958771298 | 0.605371307 | 1.019466958 | 1.119568032 | 1.17290015 | 1.613376283 |
| 4-Hydroxyphenylpropionic Acid | 2.2919747 | 1.6901954 | 1.8474124 | 5.220563295 | 5.053026598 | 6.243749347 | 2.757999676 | 2.392286847 | 3.039677402 |
| 9,12-(Z,Z)-Octadecadienoic acid | 5.728547 | 5.3229357 | 5.3284368 | 4.206471354 | 3.681251329 | 5.182497407 | 5.250892983 | 5.018663323 | 7.283700182 |
| Adenine | 2.6726627 | 2.2468416 | 2.8085889 | 24.65710324 | 23.26620429 | 30.57216668 | 16.69058706 | 19.50865906 | 26.86462462 |
| à-Hydroxyglutaric acid | 0.8140543 | 1.0150136 | 1.2524629 | 0.822420051 | 0.078657476 | 1.08534395 | 0.66772626 | 0.571721311 | 0.531840324 |
| à-Hydroxyisobutyric acid | 2.7141224 | 3.8323395 | 4.0020746 | 0.657043797 | 0.7644021 | 0.892804101 | 1.11038851 | 0.848524796 | 1.150977722 |
| Alanine | 637.30482 | 691.62052 | 780.3907 | 676.1689537 | 666.3821248 | 744.5892207 | 707.0348819 | 723.4681547 | 678.0068969 |
| Allantoin | 0.6868629 | 1.1612267 | 1.7430319 | 1.888062156 | 2.065681468 | 2.502624184 | 1.875429835 | 1.979812037 | 2.586200793 |
| Aminomalonic acid | 3.8791377 | 5.5873248 | 6.7107299 | 7.202088378 | 7.906801879 | 11.3487398 | 11.21414155 | 9.348793158 | 10.43335536 |
| Arabitol | 3.7515294 | 4.6112066 | 4.7188093 | 4.702652431 | 5.632321046 | 8.318099559 | 21.04335595 | 18.11369246 | 25.16219172 |
| Aspartic acid | 147.75013 | 126.61161 | 144.7872 | 268.1317123 | 251.3915006 | 349.1350047 | 304.4686445 | 276.9744489 | 350.4430021 |
| Benzoic acid | 41.202807 | 42.598386 | 47.793211 | 57.44016131 | 52.58597128 | 60.65490982 | 53.17198618 | 48.39279617 | 65.88477686 |
| beta-Alanine | 1.5439781 | 1.6762771 | 1.7391182 | 1.386524062 | 1.775659446 | 3.200814163 | 3.991051285 | 3.191849735 | 4.96475612 |
| Cadaverine | 148.17005 | 220.70597 | 206.7152 | 14.39966204 | 22.67988704 | 29.35089112 | 96.54495588 | 77.50549657 | 129.1428536 |
| Cellobiose | 1.8205915 | 1.1366561 | 1.1281757 | 1.439487778 | 1.956474333 | 2.539199666 | 2.330239968 | 1.600203906 | 1.673080603 |
| D-Glucuronic acid | 0.6963906 | 0.4127832 | 0.0957179 | 0 | 0.081595753 | 0.506332803 | 0 | 0.44209775 | 3.687451575 |
| Dodecyl acrylate | 2.1635247 | 2.6191343 | 3.185653 | 3.159309045 | 3.049882714 | 2.589124742 | 2.901191008 | 1.931349231 | 2.001734874 |
| Eicosanoic acid | 2.2761171 | 2.3286311 | 2.6804301 | 2.314871546 | 2.078823334 | 1.887844904 | 2.163188285 | 2.027716396 | 3.044898119 |
| Eicosanol | 1.0209258 | 0.9660479 | 1.0568506 | 1.204631296 | 1.265519354 | 1.359376514 | 0.96273932 | 1.133589655 | 1.380649588 |
| Erythritol | 9.8116778 | 9.9323497 | 11.599681 | 9.65799435 | 9.962334036 | 11.86232415 | 35.38708407 | 35.1741152 | 46.65286768 |
| Erythronic acid | 3.2362751 | 3.1974179 | 3.7327272 | 3.05676788 | 2.99757912 | 3.872379113 | 3.573018043 | 2.724462627 | 4.089190175 |
| Ethanolamine | 128.43979 | 139.32933 | 156.58521 | 179.4062044 | 170.6294233 | 124.0016533 | 113.6106956 | 133.8968568 | 106.8012257 |
| Ethyl hexadecanoate | 0.3998631 | 0.534315 | 0.4201548 | 0.284911817 | 0.28088627 | 0.286049046 | 1.413565157 | 1.639475149 | 3.0265556 |
| Ethyl linoleate | 0.8385413 | 0.8690841 | 0.7319285 | 0.592128387 | 0.711276626 | 0.713171328 | 0.794189109 | 0.641516606 | 0.986978513 |
| Ethyl linolenate | 0.1992089 | 0.3519094 | 0.3067061 | 0.072336577 | 0.139678354 | 0.152235926 | 1.239769709 | 1.055304862 | 1.683617836 |
| Ethylene glycol | 17.71751 | 32.385616 | 27.097806 | 27.2044828 | 27.38647814 | 25.90226795 | 28.1036015 | 27.48917051 | 33.90033512 |
| Fucose | 1.9018568 | 1.9150555 | 2.3012905 | 1.973822421 | 1.857995475 | 2.202379649 | 2.281335385 | 2.157600874 | 2.882323023 |
| Galactose | 8.9197524 | 8.757808 | 19.138077 | 5.371639426 | 8.979570286 | 5.31329112 | 4.633047258 | 3.951065996 | 5.053072925 |
| Glucuronic acid | 2.3824302 | 2.0674168 | 1.7452242 | 3.056069379 | 3.560821707 | 5.403373513 | 4.105596835 | 3.234612843 | 4.511199468 |
| Glutamic acid | 68.035474 | 72.560408 | 89.985932 | 38.53337109 | 34.74411184 | 44.99538363 | 27.72534162 | 24.78617051 | 33.2746492 |
| Glyceric acid | 2.5333139 | 2.2646392 | 2.6964611 | 1.302866996 | 1.356255441 | 1.716583216 | 1.756064862 | 1.498184288 | 2.14065395 |
| Glycerol | 187.06384 | 190.24901 | 193.47082 | 164.2632606 | 174.3769043 | 213.5919924 | 267.9835216 | 281.3461653 | 354.5871957 |
| Glycine | 5.7472927 | 7.4686498 | 6.6943857 | 6.509955027 | 9.13998261 | 16.30182097 | 17.62655917 | 12.3636299 | 16.94387008 |
| Heptadecanoic acid | 1.9845497 | 1.9196547 | 1.9403791 | 2.043156427 | 1.82268536 | 1.840275197 | 2.120993903 | 2.391455686 | 3.202199735 |
| Hexadecanoic acid | 185.83392 | 178.55856 | 184.17472 | 196.2916699 | 183.5808356 | 185.8725163 | 197.7215751 | 195.3542311 | 227.3400178 |
| Hexanoic acid | 1.0824131 | 1.3714536 | 1.692057 | 2.141944998 | 2.1626248 | 1.913192684 | 1.394180143 | 1.151391312 | 1.598199179 |
| Homoserine | 317.56655 | 348.97811 | 387.77693 | 437.8204521 | 411.5546544 | 309.3620697 | 277.0907809 | 326.2806633 | 257.3877629 |
| Inositol | 33.564742 | 30.286344 | 31.374813 | 38.78830796 | 38.76407121 | 48.59723636 | 39.86536498 | 34.84828206 | 48.07857325 |
| Isoleucine | 0.0964765 | 0.0919607 | 0.1189337 | 0.090745734 | 0.109314909 | 0.108046022 | 0.111612946 | 0.114014129 | 0.07797952 |
| Isovanillic Acid | 1.2588335 | 1.1412369 | 1.2864753 | 1.33224636 | 1.267566905 | 1.495603753 | 1.420687112 | 1.358151214 | 1.763236926 |
| Ketomalonic acid | 0.7405416 | 0.3221967 | 0.3432486 | 4.234174518 | 3.670494599 | 4.255038664 | 2.021111376 | 1.805536937 | 1.510987485 |
| Lactic acid | 130.91885 | 130.62014 | 134.4648 | 162.0361892 | 160.4200036 | 178.5803552 | 117.6016226 | 106.4077935 | 136.8127092 |
| Leucine | 917.0859 | 922.39284 | 905.15504 | 919.1719579 | 900.57497 | 869.7337682 | 807.5963719 | 869.7878719 | 783.6559506 |
| Linoleic acid ethyl ester | 0.2045643 | 0.2037175 | 0.1154385 | 0.030556717 | 0.090114017 | 0.092528355 | 0.527905951 | 0.561336815 | 0.804368751 |
| Linolenic acid | 37.161999 | 33.244829 | 33.091862 | 27.25596407 | 26.64925714 | 35.84874868 | 33.80706427 | 35.54359153 | 45.15484055 |
| Lysine | 9.7949889 | 11.498727 | 15.175376 | 26.7205692 | 31.04452301 | 57.02687407 | 42.74783272 | 37.87784143 | 70.09855021 |
| Malic acid | 0.5041105 | 0.1766186 | 0.2310464 | 0.183249766 | 0.05210276 | 0.307113531 | 0.27221033 | 0.141494475 | 0.495537568 |
| Malonic acid | 3.1683089 | 1.5090691 | 1.7940725 | 11.55937092 | 10.13982838 | 10.9935294 | 7.312723128 | 6.45492767 | 9.277413954 |
| Maltose | 0.6706912 | 0.615261 | 0.6161735 | 0.383028261 | 0.314601735 | 0.471705121 | 0.501549957 | 0.563388499 | 0.863658596 |
| Mannitol | 0.316454 | 0.5255876 | 0.4594122 | 0.334993908 | 0.690428111 | 0.372583945 | 3.146635108 | 2.989373203 | 5.691750391 |
| Melezitose | 1.5677217 | 0.4870693 | 0.1692712 | 0.135441565 | 0.25224058 | 0.175900224 | 0.956029123 | 0.84814002 | 0.938448866 |
| Methyl linolenate | 0.0361717 | 0.1027086 | 0.0962073 | 0.14072008 | 0.056691447 | 0.106854685 | 0.141982801 | 0.043157658 | 0.213966393 |
| Monopalmitin | 2.6293761 | 1.9270426 | 1.9068881 | 2.301540107 | 2.69476737 | 2.841630663 | 2.27882427 | 1.960165989 | 1.88059824 |
| Neophytadiene | 2.3352093 | 1.7641895 | 1.4865963 | 2.041637702 | 1.76941102 | 1.662008635 | 1.50840261 | 1.390939126 | 1.549693008 |
| N-Methoxy-Amine | 3972.1967 | 3645.8048 | 3324.0894 | 3653.13389 | 3716.342221 | 3025.424155 | 3091.190574 | 3097.382439 | 2205.630186 |
| Ononitol | 11.699399 | 12.129007 | 13.342493 | 11.44366026 | 12.41822576 | 14.87175376 | 13.88623106 | 12.88547594 | 17.12894147 |
| Ornithine | 20.272357 | 30.159841 | 36.052775 | 31.03107763 | 36.57828519 | 65.58863513 | 60.20032311 | 47.77494474 | 89.55424061 |
| p-Coumaric acid | 61.867557 | 9.2020519 | 6.3217983 | 5.682630738 | 4.31272301 | 3.387484999 | 4.303174862 | 3.649652183 | 4.249683557 |
| Pentitol | 1.3357578 | 1.2582545 | 1.5577242 | 1.497141648 | 1.258732379 | 0.975122902 | 1.844160992 | 2.301524839 | 2.885244536 |
| Phenethylamine | 3.0224459 | 3.6096643 | 4.2532371 | 0.247467238 | 0.294867865 | 0.281681099 | 1.311828106 | 0.987440353 | 1.183282486 |
| Phenol | 2.102062 | 2.5160685 | 4.0866358 | 4.365659316 | 4.286843234 | 3.575995112 | 4.002085832 | 3.121793523 | 4.302110148 |
| Phenylalanine | 11.36425 | 12.793308 | 15.664165 | 16.46495718 | 16.51120508 | 20.88368122 | 20.07121647 | 20.37365107 | 27.10936034 |
| Phenyllactic acid | 9.6945437 | 11.043603 | 11.886264 | 12.89127426 | 11.8001523 | 15.09272953 | 5.288629442 | 4.928200128 | 6.767342711 |
| Phytol | 66.917759 | 53.290556 | 47.315404 | 60.76411025 | 47.57525441 | 99.39503026 | 94.51933973 | 103.5010241 | 213.009018 |
| Pinitol | 569.32335 | 539.08206 | 547.92365 | 564.038514 | 561.3330888 | 608.7989466 | 562.7968222 | 579.6628674 | 617.099618 |
| Pipecolic acid | 45.360103 | 50.369439 | 58.450211 | 51.3101165 | 54.84119054 | 65.43130542 | 59.909531 | 57.91981482 | 78.10236819 |
| Proline | 816.8485 | 873.14113 | 882.13054 | 877.9675341 | 845.8202541 | 869.1641044 | 862.7367086 | 869.6926894 | 836.3473924 |
| Pseudouridine | 0.0844412 | 0.0513658 | 0.1683258 | 0.16936934 | 0.17678398 | 0.20747987 | 0.212400534 | 0.175257943 | 0.344169612 |
| Putrescine | 6.4597495 | 7.8019887 | 7.5632935 | 7.981159851 | 9.088595815 | 8.374393768 | 8.805369201 | 8.101785674 | 6.527405725 |
| Ribonic acid | 2.0728564 | 0.2547649 | 2.8502976 | 1.717412522 | 1.749363987 | 2.205346648 | 2.749895858 | 1.874427664 | 3.45977137 |
| Salicylic acid | 55.43849 | 54.080252 | 61.370775 | 63.41845765 | 60.7588155 | 73.83615688 | 64.66970915 | 66.66366502 | 79.73457952 |
| Sedoheptulose | 7.3036565 | 7.3851297 | 7.631413 | 7.967191634 | 8.069178851 | 10.50993406 | 9.179350854 | 8.215151951 | 11.64514311 |
| Serine | 1.0511556 | 5.8273222 | 4.7156559 | 5.048399034 | 6.689135184 | 8.98163079 | 5.620968924 | 5.829276956 | 9.035866935 |
| Stearic acid | 53.824645 | 56.449013 | 59.210796 | 56.63775933 | 59.11427458 | 50.80804438 | 57.24725203 | 60.30960572 | 56.56626416 |
| Succinic acid | 132.29864 | 156.61427 | 185.93421 | 79.99345017 | 77.1430661 | 91.74779793 | 108.8089779 | 99.57740415 | 126.7967797 |
| Sucrose | 9.9861098 | 69.227288 | 2.7041399 | 1.917466978 | 3.431171946 | 7.678544515 | 1.581866842 | 1.514571198 | 1.08448892 |
| Tetradecanoic acid | 3.1313666 | 3.4327397 | 3.4373748 | 2.781403723 | 2.826286732 | 3.341512926 | 3.226558785 | 11.82496677 | 10.92582031 |
| Threitol | 1.3575908 | 1.5170697 | 1.656642 | 1.88202847 | 1.827048079 | 2.269155229 | 2.894530516 | 2.902064497 | 3.924898958 |
| Threonic acid | 10.789228 | 10.120437 | 11.512504 | 9.817020267 | 9.493885759 | 12.67473046 | 10.87595461 | 8.960515675 | 13.48653807 |
| Threonine | 41.087717 | 38.051525 | 44.716862 | 59.96543896 | 59.61976245 | 74.21121925 | 68.64651911 | 64.52372823 | 88.71602227 |
| Thymine | 16.242177 | 16.314022 | 19.712548 | 15.82802393 | 15.23554858 | 19.22480741 | 18.65772797 | 20.3032853 | 26.07050533 |
| trans-Ferulic acid | 6.1982557 | 2.6978373 | 2.8407106 | 1.833750762 | 1.529881402 | 1.708046398 | 4.535396583 | 4.391266712 | 7.091033725 |
| Tryptophan | 47.364778 | 52.127349 | 54.316426 | 46.04312421 | 45.82456281 | 47.32475085 | 55.16646091 | 46.96212744 | 54.10942459 |
| Tyramine | 285.59872 | 322.42814 | 328.69501 | 234.5432823 | 256.0530732 | 334.327107 | 378.9096241 | 324.7260155 | 390.3332797 |
| Tyrosine | 2.1634732 | 2.732669 | 4.7899707 | 69.46226277 | 78.37776755 | 117.3051773 | 65.83776778 | 86.7278986 | 142.7114368 |
| Uracil | 34.541531 | 35.469117 | 43.716919 | 34.76160107 | 33.44948246 | 41.76704828 | 45.9405722 | 47.33215132 | 61.25641159 |
| Valine | 146.64971 | 158.29809 | 181.37449 | 160.9299157 | 160.9608047 | 195.4661128 | 201.1071804 | 202.5091398 | 255.6293424 |
| Vanillylpropionic acid | 14.388157 | 14.370259 | 14.667354 | 20.50491457 | 19.24096883 | 24.98739547 | 17.73738716 | 17.45530318 | 21.58848196 |
| Viburnitol | 0.2634116 | 0.2225107 | 6.9108344 | 0.686473107 | 0 | 0.660597809 | 8.544229791 | 4.67844241 | 0.131405489 |
| Xylitol | 3.3721668 | 3.138066 | 3.7513506 | 2.955624701 | 3.084965022 | 3.743722447 | 4.03822071 | 3.824103788 | 5.091382864 |
| Xylonic acid-lactone | 3.5791006 | 2.6241291 | 3.1380594 | 4.207482112 | 3.874336502 | 4.647233182 | 3.009185746 | 2.856362668 | 3.738313337 |
| Xylose | 4.6654992 | 3.2499713 | 3.381612 | 2.802913483 | 3.081938441 | 3.873069771 | 3.664239832 | 3.735667318 | 5.441131011 |
| Xylulose | 115.35212 | 121.19316 | 133.25037 | 117.5002072 | 118.9837579 | 152.6228433 | 145.6667773 | 115.2994271 | 178.1620648 |

^1^The relative concentration of 102 indentified metabolites was the total mass of the signal integration area, and was normalized to 10,000 for each sample. Metabolites annotation was performed by importing the normalized data into the Simca-P software (version 11.5) to detect differentially expressed metabolites, and the NIST (http://www.nist.gov/index.html) and KEGG (http://www.genome.jp/kegg/) commercial databases were used to search for metabolites.
